# Branched-chain ketoacids derived from cancer cells modulate macrophage polarization and metabolic reprogramming

**DOI:** 10.3389/fimmu.2022.966158

**Published:** 2022-10-13

**Authors:** Zhengnan Cai, Wan Li, Martin Brenner, Sheyda Bahiraii, Elke H. Heiss, Wolfram Weckwerth

**Affiliations:** ^1^ Molecular Systems Biology (MOSYS), Department of Functional and Evolutionary Ecology, University of Vienna, Vienna, Austria; ^2^ Department of Pharmaceutical Sciences, University of Vienna, Vienna, Austria; ^3^ Vienna Metabolomics Center (VIME), University of Vienna, Vienna, Austria

**Keywords:** macrophage polarization, tumor-associated macrophages, panomics, BCKAs Fc-gamma receptor (FCgR)-mediated phagocytosis, tumor necrosis factor alpha (TNFα)-nuclear factor kappa B (NFκB) -mediated inflammatory pathway, apoptosis

## Abstract

Macrophages are prominent immune cells in the tumor microenvironment that can be educated into pro-tumoral phenotype by tumor cells to favor tumor growth and metastasis. The mechanisms that mediate a mutualistic relationship between tumor cells and macrophages remain poorly characterized. Here, we have shown *in vitro* that different human and murine cancer cell lines release branched-chain α-ketoacids (BCKAs) into the extracellular milieu, which influence macrophage polarization in an monocarboxylate transporter 1 (MCT1)-dependent manner. We found that α-ketoisocaproate (KIC) and α-keto-β-methylvalerate (KMV) induced a pro-tumoral macrophage state, whereas α-ketoisovalerate (KIV) exerted a pro-inflammatory effect on macrophages. This process was further investigated by a combined metabolomics/proteomics platform. Uptake of KMV and KIC fueled macrophage tricarboxylic acid (TCA) cycle intermediates and increased polyamine metabolism. Proteomic and pathway analyses revealed that the three BCKAs, especially KMV, exhibited divergent effects on the inflammatory signal pathways, phagocytosis, apoptosis and redox balance. These findings uncover cancer-derived BCKAs as novel determinants for macrophage polarization with potential to be selectively exploited for optimizing antitumor immune responses.

## Introduction

Over the past decade, a wealth of preclinical and clinical evidence supports a tumor-promoting role for macrophages in cancer (1). For example, macrophage contents in solid tumors correlate with chemotherapy resistance and a worse prognosis for non-small cell lung carcinoma (NSCLC), breast cancer, pancreatic cancer, glioblastoma, and lymphoma (2–6). Indeed, macrophages were thought to be antitumoral at an early stage of tumor onset, owing to their ability to directly engulf and kill tumor cells or indirectly clear tumor cells through presenting tumor antigens to activate cytotoxic lymphocytes, but once macrophages were influenced by cancer cells, they rapidly adopt an alternative phenotype with an immunosuppressive function that enhances tumor progression and metastasis. Even though there may still be antitumor macrophages present, most macrophages are “educated” to favor tumor growth (7). Thus, understanding how cancer cells govern macrophage phenotype within the tumor microenvironment provides a means to selectively target macrophage reprogramming and improve tumor immune surveillance.

Cancer cell-secreted metabolites within the tumor microenvironment are partially responsible for modulating surrounding immune profiles (8). Lactate is largely produced within the tumor microenvironment by cancers cells exploiting the Warburg effect (aerobic glycolysis) (9), which promotes differentiation and polarization of tumor-associated macrophages towards a pro-tumoral phenotype (M2-like) with elevated expression of arginase-1 (ARG1), pro-tumoral marker *Vegf* and M2 marker genes (*Arg1, Fizz1, Mgl1, Mgl2*) (10). In turn, M2-like macrophages produce immunosuppressive cytokines (IL10) and metabolites such as arginine, ornithine and polyamines, which are essential for the cell division and proliferation of some tumors (11, 12). In addition, tumor cell-derived kynurenine dampened the effector function of macrophages and engaged a pro-tumoral cooperation between macrophages and regulatory T cells (Tregs) (13). TCA cycle intermediate succinate released by lung cancer cells can activate macrophage polarization into an M2-like phenotype, and accelerate cancer cell migration and metastasis (14).

Our initial unpublished metabolomic analysis showed elevated BCKAs levels in the conditioned media (CM) from two lung cancer cell lines (A549 and AE17) compared to non-proliferative cells. Recently, accumulated BCKAs were also found in glioblastoma and can act as substrates for *de novo* synthesis of branched-chain amino acids (BCAAs) in macrophages (15). The three BCKAs are α-ketoisocaproate (KIC), α-keto-β-methylvalerate (KMV), and α-ketoisovalerate (KIV). All are precursors for or generated from essential amino acids leucine, isoleucine, and valine. The reaction is catalyzed by the compartment-specific BCAAs transaminases (cytoplasmic, BCAT1; mitochondrial, BCAT2). Within cells or tissues, BCKAs can either be reversibly transaminated to BCAAs and α-ketoglutarate (α-KG) or catabolized to branched-chain acyl-CoA (R-CoA) that can be further metabolized by multimeric BCKA dehydrogenase enzyme complex (BCKDH) to generate a branched-chain acyl-CoA (R-CoA) that can be further metabolized to acetyl-coenzyme A (acetyl-CoA) or succinyl-CoA, which finally fuel the tricarboxylic acid (TCA) cycle (16) (**Figure S1A**). BCAT1 and BCAT2 are highly expressed in human tumors such as glioblastoma, acute myeloid leukaemia, lung tumor, breast tumor and pancreatic tumor (17–22). BCAT1 inhibition significantly reduced BCKAs within pancreatic stromal cells (22). Notably, uptake of BCKAs suppressed the phagocytosis of macrophages suggesting an immunosuppressive function of these metabolites (15). In contrast, BCKAs induced upregulation of proinflammatory genes and inflammation-related cytokines in bone marrow-derived macrophages (BMDM) from wild-type and db/db mice (type 2 diabetes) (23). Thus, how BCKAs modulate macrophage phenotype remains controversial and underlying mechanisms and context-dependency are far from being completely understood.

Here, we uncover the role of BCKAs in regulating macrophage activation and metabolic reprogramming. We found that a panel of selected cancer cells secrete all three BCKAs to different extents, of which KIV exhibited an effect on pro-inflammatory (M1-like) macrophage polarization while KIC and KMV showed an effect on pro-tumoral (M2-like) macrophage polarization. Combined BCKAs promoted M2-like polarization and was closer to tumor setting. Proteomic analyses revealed that KMV stimulation affected Fc-gamma receptor (FcγR)-mediated phagocytosis, tumor necrosis factor-alpha (TNFα)-nuclear factor kappa B (NFκB)-mediated inflammatory pathway and apoptosis. Further metabolomics analyses indicated that cancer-derived BCKAs were used for augmenting TCA cycle intermediates. KIC and KMV also contributed to enhanced polyamine metabolism in macrophages. Hence, preventing the release of BCKAs by cancer cells, and selectively targeting KMV or KIC in the tumor microenvironment would benefit antitumor immunity.

## Results

### Cancer cell-derived BCKAs modulate macrophage polarization *via* MCT1

**Figure 1:**
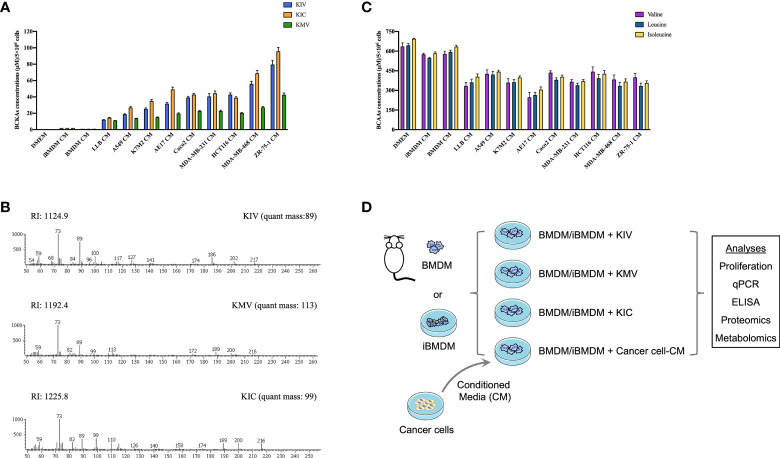
Cancer cells consume BCAAs and secrete BCKAs. **(A)**, BCKAs (KIV, KIC and KMV) in the DMEM complete medium or conditioned media (CM) from indicated cells were detected by GC-MS. **(B)**, Representative mass spectra of KIV, KIC and KMV by GC-MS. **(C)**, BCAAs (leucine, isoleucine and valine) in the DMEM complete medium or conditioned media from indicated cells were detected by GC-MS. **(D)**, Diagram of workflow. Conditioned media from cancer cells and three BCKAs were added to BMDM or iBMDM, followed by multiomics analysis. Data in A and C show the mean ± SEM of n = 3 technical experiments.

**Figure 2:**
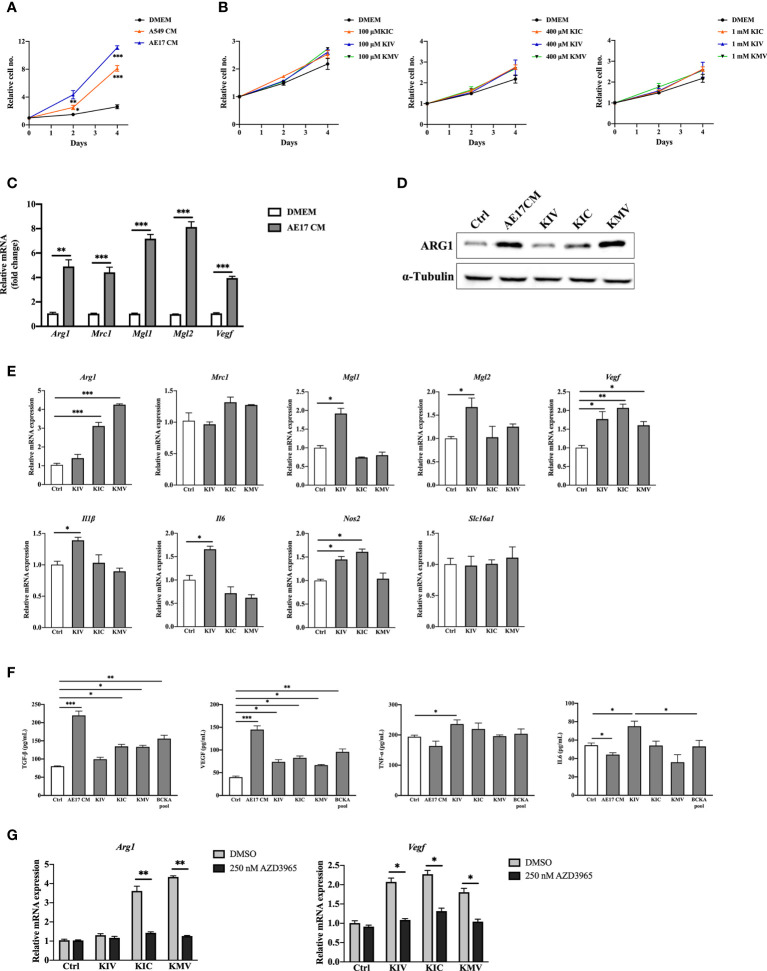
BCKAs in cancer cell CM induce murine BMDM polarization. **(A)**, Relative proliferation rate of BMDM grown in DMEM complete medium or indicated cancer cell CM. **(B)**, Relative proliferation rate of BMDM grown in DMEM complete media or equivalent media containing 200 μM KIV or 200 μM KIC or 200 μM KMV (n = 3). **(C)**, mRNA expression of *Arg1*, *Mrc1*, *Mgl1* and *Mgl2* in BMDM was measured by qPCR after incubation with AE17 CM for 24 hours. **(D)**, ARG1 protein level was measured by western blot after incubation with AE17 CM for 24 hours. **(E)**, mRNA expression of *Arg1*, *Mrc1*, *Mgl1*, *Mgl2*, *Vegf*, *Il1β*, *Il6*, *Nos2* and *Slc16a1* was measured in BMDM after stimulation with DMEM (Control), 200 μM KIV, 200 μM KIC and 200 μM KMV, respectively for 24 hours. **(F)**, Cytokines TGF-β, TNF-α, IL6 and growth factor VEGF in BMDMs after the indicated treatments were measured by ELISA kits. **(G)**, mRNA expression of *Arg1* and *Vegf* was measured after pretreatment with the MCT1 inhibitor 250 nM AZD3965 for 1 hour, and stimulation with 200 μM KIV, 200 μM KIC and 200 μM KMV, respectively for 24 hours. Data show the mean ± SEM of n = 3 biological experiments. *p < 0.05, **p < 0.01, ***p < 0.001 (unpaired two-tailed t test).

To examine whether cancer cell-derived BCKAs can modulate macrophage activation, we first measured concentrations of KIV, KIC and KMV in the conditioned media (CM) from 9 equally seeded cancer cell lines (6 human cancer cell lines and 3 murine cancer cell lines), as well as immortalized bone marrow derived-macrophages (iBMDM) and BMDM. Both iBMDM and BMDM consumed a few BCAAs and released a low quantity of BCKAs (**Figures 1A, B**). In contrast, accumulated BCKAs were detected in cancer CM, which is consistent with the consumption of BCAAs by cancer cells (**Figure 1C**). The contents of the three BCKAs varied substantially in each cancer cell CM, where KIV ranges from 15 μM to 80 μM, KIC ranges from 15 μM to 100 μM and KMV ranges from 10 μM to 40 μM. To keep our initial findings consistent, we evaluated the effect of BCKAs and two lung cancer CM (A549 CM and AE17 CM) on macrophage proliferation and polarization (**Figure 1D**). Both BMDM and iBMDM proliferation rates were significantly enhanced on exposure to A549 CM and AE17 CM (**Figures 2A**, **S1B**), probably because of cancer cell-released various cytokines (e.g., macrophage colony-stimulating factor (M-CSF/CSF-1)) and soluble factors that affect macrophage differentiation and proliferation. While stimulation with BCKAs only had little effect on macrophage proliferation even at a concentration of 1 mM (**Figures 2B**, **S1C**). Moreover, both iBMDM and BMDM exhibited an increase in expression of (Arginase-1) ARG1 protein and M2 marker genes *Arg1, Mgl1, Mgl2, Mrc1* and a pro-tumoral marker *Vegf* after AE17 CM treatment (**Figures 2C, D**, **S1D, E**). BMDM also significantly secreted anti-inflammatory cytokine TGF-β and growth factor VEGF after AE17 CM treatment, whereas proinflammatory cytokines TNF-α and IL6 were slightly reduced (**Figure 2F**). However, the individual BCKAs differed in their activation of genes and cytokines associated with immune suppression, chronic inflammation, and tumor angiogenesis in macrophages. *Vegf* mRNA expression was increased in both iBMDM and BMDM on stimulation with all three BCKAs, while *Arg1* was only enhanced by KIC and KMV stimulation (**Figures 2E**, **S1F**). *Mgl1* and *Mgl2* were solely enhanced by KIV stimulation (**Figure 2E**). Both KIV and KIC additionally enhanced the proinflammatory (M1-like) marker *Nos2*, while only KIV stimulation also activated *Il1β* and *Il6* (**Figures 2E**, **S1F**). KIC and KMV treatment also caused the production of anti-inflammatory cytokine TGF-β and growth factor VEGF (**Figure 2F**). Similar effects on TGF-β and VEGF production by BCKA pool treatment were observed (**Figure 2F**). Proinflammatory cytokines IL6 and TNF-α were only increased after KIV treatment while slightly reduced in KMV-treated macrophages (**Figure 2F**). Interestingly, BCKA pool treatment did not affect TNF-α and modestly reduced IL6 production, which is closer to AE17 CM treatment and probably due to the opposing effects from the other two BCKAs, especially KMV.

The monocarboxylate transporter MCT1 mediates the transport of BCKAs through plasma membranes of Xenopus oocytes and glioblastoma cells (15, 24, 25). We thus investigated whether BCKAs activate macrophage MCT1 expression. RT-quantitative PCR (qPCR) analyses of BMDM showed that BCKAs stimulation has no effect on MCT1 (*Slc16a1*) mRNA level (**Figure 2E**). However, BCKAs-induced upregulation of *Arg1* and *Vegf* was significantly reduced by the MCT1 inhibitor AZD3965 (**Figure 2G**). We repeated the same experiments with iBMDM and found similar results (**Figure S1G**). Taken together, these data indicate that cancer cell-secreted KIV promote pro-inflammatory macrophage polarization whereas KIC and KMV promote a pro-tumoral phenotype (based on the investigated markers). Although individual BCKAs differed in macrophage polarization, combined BCKAs have a crucial impact on macrophage pro-tumoral polarization and all three BCKAs seem to affect macrophage polarization only after import by MCT1.

### Macrophage apoptosis, redox balance and inflammatory functions are significantly altered by KMV stimulation

**Figure 3:**
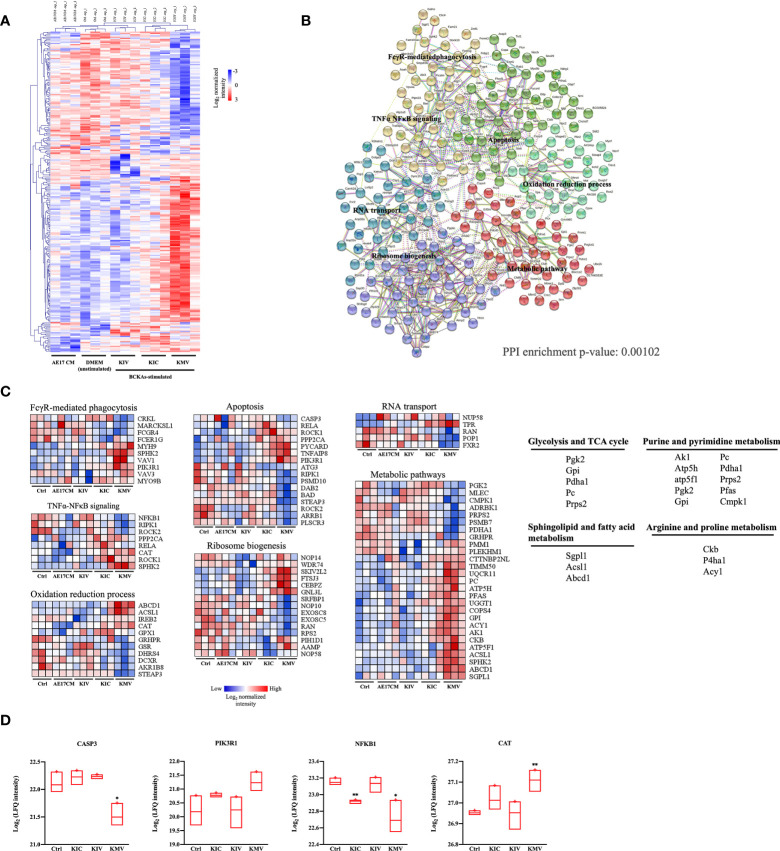
Protein signatures of responses to BCKAs stimulation. **(A)**, Heatmap of 243 differentially expressed proteins (one-way ANOVA, FDR q value < 0.05) between unstimulated (DMEM) and three BCKAs-stimulated iBMDM. **(B)**, Protein–protein interaction (PPI) network in differentially expressed proteins shown in A illustrated by STRING (PPI enrichment p-value = 0.00102). Proteins were divided into 6 clusters by k-means clustering method. Each node represents input proteins. Distinct nod colors indicated 1-6 clusters (see **Table S1**). Edges represent protein-protein associations (evidence). Representative KEGG terms are shown in enriched positions. **(C)**, Heatmap of the protein intensity of selected KEGG terms shown in **(B, D),** Proteomics analysis of CASP3, PIK3R1, NFKB1 and CAT in Ctrl and three BCKAs-stimulated iBMDM. The LFQ intensities were log2 transformed. Data shown the mean ± SEM of n = 3 biological experiments. *p < 0.05, **p < 0.01 (unpaired two-tailed t test).

To investigate the global responses of macrophages to BCKAs, we performed an untargeted MS-based proteomic analysis on iBMDM treated with DMEM (Ctrl), AE17 CM, 200 μM KIC, 200 μM KIV and 200 μM KMV, respectively. Overall, 3512 protein groups were quantified (**Table S1**) upon data processing (see methods). Next we applied one-way analysis of variance (ANOVA) with an False Discovery Rate (FDR) cutoff (FDR value < 0.05) and found 114 and 130 differentially expressed proteins (DEPs) in the DMEM group and in the combined three BCKAs groups, respectively (**Table S1**, **Figure 3A**). Principal component analysis (PCA) reliably distinguished DEPs from three BCKAs groups versus the control group (**Figure S2A**). Functional analysis indicated that the most enriched categories of BCKAs-upregulated proteins included the cAMP signaling pathway, sphingolipid signaling pathway, autophagy, VEGF signaling pathway, nucleotide synthesis and mitochondrial transport (**Figure S2B**). Among the top categories of suppressed proteins were RNA splicing, regulation of cytochrome c release, apoptosis, RNA destabilization and TNFα-NFκB pathway (**Figure 2B**).

Next, we used all DEPs to construct a protein interaction map. Most of these proteins exhibited significant interactions (PPI = 0.00102) (**Table S1**, **Figure 3B**). 6 clusters of DEPs were identified by way of k-means analysis and enriched in canonical macrophage functions (**Figures 3B, C**), including FcγR pathway-mediated phagocytosis (FCGR4, SPHK2, PIK3R1), apoptosis (CASP3, STEAP3, ROCK1, ROCK2, RIPK1, PIK3R1), TNFα-NFκB signaling (NFKB1, RELA, RIPK1, PPP2CA, SPHK2), oxidation-reduction process (CAT, GSR, GPX1), ribosome biogenesis (NOP58, NOP10, NOP14, WDR74), RNA transport (POP1, RAN, NUPL1, TPR, FXR2) and metabolic pathways (CMPK1, GRHPR, GPI, ACSL1, ABCD1, CKB, P4HA1, PFAS, AK1, SGPL1, ATP5H). Of note, KMV stimulation has more obvious alterations in these pathways than KIC and KIV. Furthermore, we compared the top26 node degree of proteins (each protein interacts with at least 10 other proteins) among control and three BCKAs groups (**Table S1**). We found that apoptosis executor CASP3 was reduced while negative apoptosis regulator PIK3RL was increased by KMV stimulation (**Figure 3D**), suggesting that KMV stimulation protects macrophages from apoptosis. Similarly, reduced inflammatory regulator NFKB1 and increased negative regulator CAT indicated that KMV stimulation prevented macrophage polarizing into inflammatory tumor suppressive phenotype (**Figure 3D**), which is in line with our previous qPCR results. In addition, KMV stimulation changed several metabolic pathways such as glycolysis, TCA cycle, purine and pyrimidine nucleotide synthesis, arginine and proline metabolism and sphingolipid metabolism (**Figure 3C**).

### BCKA stimulation elevates metabolites in the TCA cycle and polyamine metabolism

**Figure 4:**
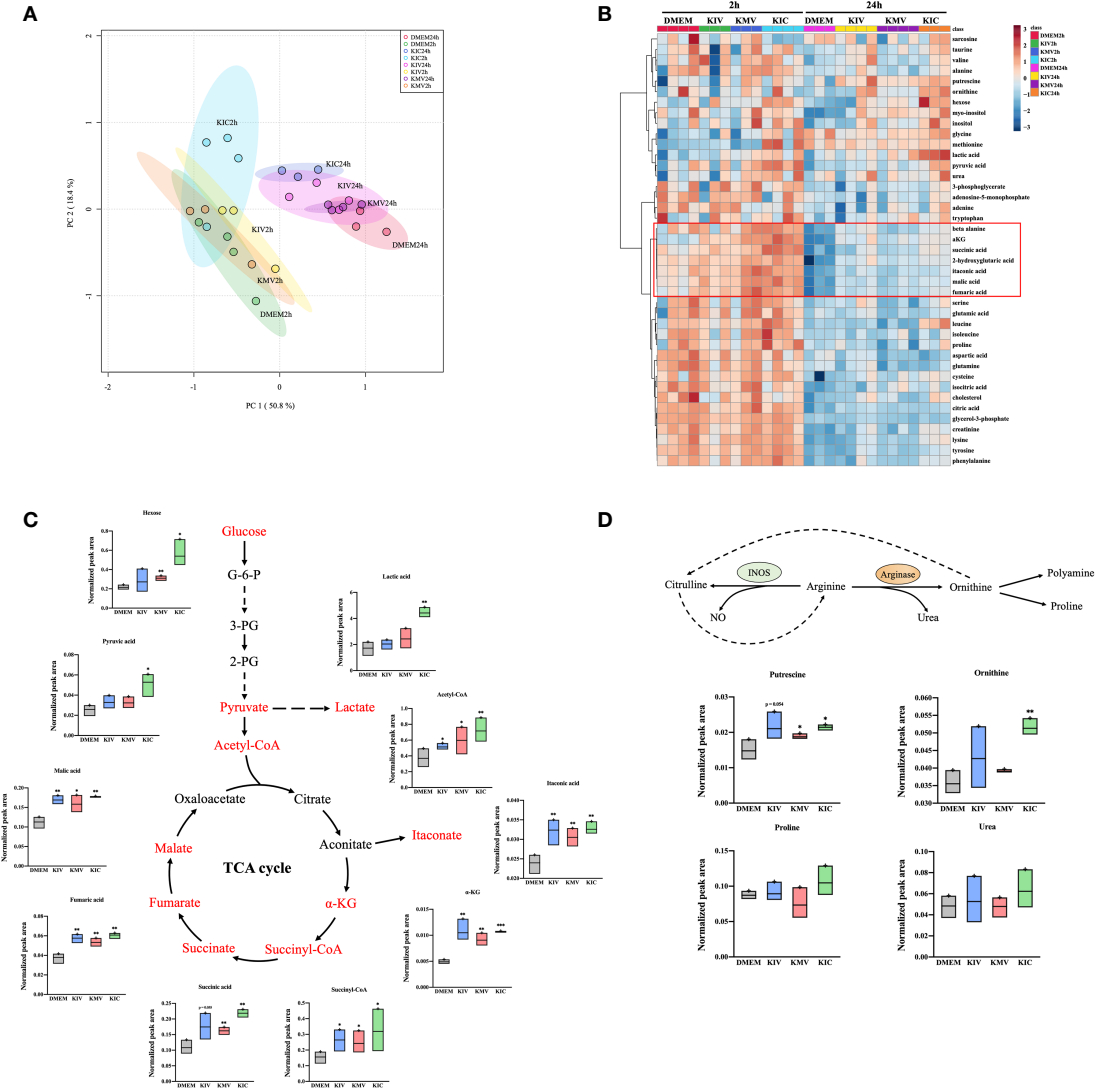
BCKAs stimulation enhance TCA cycle and polyamine metabolism in macrophages. **(A)**, PCA analysis of total identified metabolites among DMEM, KIV, KMV and KIC treatment at two time points (2 h and 24 h) is shown. **(B)**, The hierarchal clustering heatmap of identified metabolites upon DMEM and three BCKAs treatment at two time points (2 h and 24 h) is shown. The rows indicate different metabolites, and the columns indicate different conditions. The log-transformed metabolite intensities (normalized to internal standards) were scaled by autoscaling (mean-centering/standard deviation). **(C)**, Schematic representation of the TCA cycle, the corresponding metabolite is shown. **(D)**, Schematic representation of the arginine metabolism at 24 h, the corresponding metabolites from **(B)** are shown. PCA **(A)** and hierarchical clustering **(B)** analysis were applied *via* online tool MetaboAnalyst. Data shown the mean ± SEM of n = 3 or 4 biological experiments. *p< 0.05, **p < 0.01, ***p < 0.001 (unpaired two-tailed t test) (unpaired two-tailed t test).

Metabolic reprogramming of macrophages shapes their activation state and function. Our proteomic analysis suggested a plausible metabolic alteration in response to BCKA stimulation, we then decided to examine the metabolome of macrophages after BCKA treatment. Because macrophage metabolism is constantly changing, we harvested unstimulated and iBMDM stimulated with each of the three BCKAs at two-time points: 2 hours (h) for early response and 24 h for the late response. Totally 41 metabolites were identified by GC-MS analysis, including glycolysis, TCA cycle, amino acids metabolism, and polyamine metabolism. Clusters of 2 h groups were notably separated from 24 h by PCA plot (**Figure 4A**), indicating a dynamic metabolic change within the macrophages. An interesting trend was observed after exposure to BCKAs, where all clusters of late responses of BCKAs groups, especially KIC, have shifted away from the DMEM (control) group (**Figure 4A**). Indeed, a variety of intracellular metabolites were altered after stimulation with BCKAs, and this difference was more profound at 24 h compared to the DMEM group (**Table S2**, **Figure 4B**). Evidently, TCA cycle intermediates α-ketoglutarate (α-KG), succinyl-CoA, succinate, fumarate, and malate are all enhanced by the administration of any of the three BCKAs at both time points. Meanwhile, individual BCKAs treatment significantly increased acetyl-CoA levels, implying that BCKAs can be catabolized by macrophages and contribute to the TCA cycle. Although glutamine and glutamic acid levels were not changed by individual BCKAs treatment (**Figures 4B**, **S3A**), increased α-KG may also be from the contribution of BCKA transamination. Thus, our results indicate that BCKAs are taken up and incorporated into the TCA cycle *via* acetyl-CoA, succinyl-CoA, or α-KG (**Figures 4B, C**). The glycolysis intermediates pyruvate and lactate were enhanced by KMV and KIC at 24 h. In addition, inflammatory-related metabolites itaconate, β-alanine and 2-hydroxyglutarate were enhanced by BCKAs at 24 h (**Figure 4B**). KIC and KMV also enhanced putrescine, which is the important metabolite of polyamine metabolism (**Figure 4D**). Moreover, KIC stimulation at 24 h significantly activated creatinine, the by-product of energy metabolism and other amino acids tyrosine, alanine, cysteine, phenylalanine, taurine, and lysine (**Figure 4B**). Of note, a recent study reported that macrophages take up cancer cell-released KIV, KIC and KMV for regenerating valine, leucine and isoleucine (15). However, our metabolic data only revealed an increase in leucine level in the KIC stimulation group (**Figure 4B**). One possibility is that macrophages utilize regenerated BCAAs from BCKAs instead of extracellular sources (15). Moreover, no intracellular BCKAs were observed after 2 h or 24 h treatment, it is probably because intracellular concentrations of BCKAs are too low to be detected by the mass spectrometer or BCKAs are quickly catabolized and transaminated into the TAC cycle and BCAAs, respectively.

Next, we performed pathway analysis using selected 17 metabolites at 24 h whose FDR was < 0.05 by one-way ANOVA (**Table S2**). The most enriched metabolic pathways were the TCA cycle (citrate cycle), pyruvate metabolism and alanine, aspartate and glutamate metabolism in macrophages (**Figure S3B**). Taken together, these data provide evidence that administration of BCKAs reprograms macrophage metabolic profiles.

## Discussion

The diverse metabolic environment of tumors has long been implied to influence the phenotype of tumor-associated macrophages, rendering them immunosuppressive and contributing to tumor growth and metastasis (10, 14, 26, 27). Here, we demonstrate that cancer cells secreted three BCKAs that are able to influence macrophage polarization and metabolic reprogramming. Previous work has described the importance of BCKAs in favoring tumor growth and accelerating chronic inflammatory disease (22, 23). Our work suggests that three BCKAs exhibit divergent roles in affecting macrophage functional pathways and should be targeted individually to improve anti-tumor immune responses.

Many solid tumor cells rely heavily on amino acids for their proliferation, especially on BCAAs, which they can only derive from the diet or the tumor microenvironment (28, 29). BCKAs are the intermediates of BCAAs catabolism, which can be exported into extracellular space by MCT1 in glioblastoma (15). Accumulation of BCKAs is also correlated with various diseases including obesity, type 2 diabetes and cardiac dysfunction (30–32). We showed that a panel of cancer cells including breast, colorectal and lung cancer cells consumed BCAAs from media and released high levels of KIV, KIC and KMV. KIC and KMV could induce pro-tumoral polarization of macrophages whereas KIV could exert pro-inflammatory effects on macrophages. MCT1 is well known as the lactate transporter that mediate lactate influx and efflux (33, 34). Tumor-associated macrophages highly expressed MCT1, which is correlated with poor prognosis in breast cancer patients (35). MCT1 also mediated M2-like macrophage polarization instructed by lactate within an ischemic muscle (36). We found that BCKAs influenced macrophage polarization in an MCT1-dependent manner. Considering a higher lactate level was observed in KIC-treated macrophages, our results imply that the antitumor benefits of targeting MCT1 may partially be due to the blocked uptake of cancer-derived BCKAs or KIC-triggered lactate by surrounding immune cells.

Tumor cells have evolved to evade the engulfment by macrophages *via* the expression of anti-phagocytic molecules, leading to immune escape and macrophage repolarization (37). FcγRIV (encoded by gene *Fcgr4*) was recently identified as an important Fc receptor that promotes macrophage-mediated phagocytosis, proinflammatory cytokine production and antigen presentation to T cells (38). Activated *Fcgr4* by Intravenous Igs (IVIg) treatment repolarized M2-like macrophages switch to M1-like macrophages and impaired tumor progression and metastasis (39). Our data revealed that KMV stimulation significantly increased majority of proteins (including phagocytosis negative regulator SPHK2) associated with FcγR-mediated phagocytosis pathway while significantly reduced FCGR4 protein intensity, which may partially explain why BCKAs stimulation suppressed macrophage phagocytosis (15). Transcription factor NFκB has been shown to play a critical role in regulating the expression of proinflammatory mediators, such as nitric oxide (NO) synthase and IL-1β, IL-6 and TNF-α. Interestingly, NFKB1 intensity was decreased by KMV or KIC stimulation but not KIV stimulation. We also found that catalase (CAT) and serine/threonine-protein phosphatase 2A catalytic subunit alpha isoform (PPP2CA) proteins are increased in KMV group. CAT overexpression can inhibit NFκB activation triggered by a peroxisome proliferator and protect liver cells from oxidative damage (40). Similar, PPP2CA is a vital constituent of PP2A and its downregulation by breast cancer cell-derived exosomes activated NFκB signaling pathway in tumor-associated macrophages (41). Our proteomics and qPCR data imply that KMV probably activates CAT and PPP2CA-mediated downregulation of NFκB signal transduction, thereby limiting the expression of proinflammatory genes. However, the causal relationship between this signal axis and KMV-induced macrophage polarization and the detailed experimental validation of the proteomic data remain to be addressed in the future studies.

Recent reports revealed a mutualistic relationship concerning BCAAs metabolism between tumor cells and the tumor microenvironment (15, 22). Cancer-associated fibroblasts-secreted BCKAs can be taken up by pancreatic cancer cells and re-aminated to BCAAs, which are further involved in maintaining cellular protein synthesis, fueling the TCA cycle and increasing oxidative phosphorylation (OXPHOS) (22). In contrast, we did not find an increase of all three BCAAs in macrophages except leucine after BCKAs exposure, but our data showed the enhancement of glycolysis and TCA cycle metabolites upon BCKAs stimulation. Metabolism has been highlighted as a crucial mediator of macrophage activation and polarization (42). It is well known that enhanced glycolysis and accumulated TCA cycle intermediates such as citrate and succinate emerge as typical features of M1-like macrophages (43, 44). KIV, KMV, and KIC treatment all triggered TCA cycle intermediates while only KIV activated proinflammatory cytokines IL6 and TNF-α as well as transcripts of *Il6* and *Il1β*. Combined with our proteomics results, it seems that KMV and KIC, not KIV inhibit the NFκB pathway, which is responsible for the transcription of these proinflammatory cytokines. In M2-like macrophages, arginine is metabolized to urea and polyamines (putrescine and ornithine) by highly expressed ARG1. Increased polyamines have been found to promote tumor growth and blunt effector T cell responses (45, 46). Our results found that KMV and KIC treatment significantly increased putrescine, which supports the role of KMV and KIC on macrophage pro-tumoral polarization. Moreover, mitochondrial OXPHOS and redox homeostasis are essential for both M1-like and M2-like macrophages. M2-like macrophages are crucially dependent on the efficient electron transport chain and OXPHOS to support their energy demands and phenotype, whereas M1-like macrophages shift mitochondria away from ATP production and towards ROS production, which drives IL-1β generation and undermines anti-inflammatory activation (47). Our proteomic results revealed that KIV treatment induced GSR protein expression, which catalyzes GSH synthesis and highlighted the importance of GSH in maintaining cellular redox balance during macrophage proinflammatory activation. Unlike KIV treatment, KMV treatment decreased GSR expression while enhancing another antioxidant enzyme CAT. Thus, redox regulation is associated with BCKAs-induced macrophage polarization, and the complex mechanism remains further investigated.

In summary, we have shown that cancer cells secrete BCKAs into the extracellular milieu, which can affect distinct macrophage polarization by altering proinflammatory and anti-inflammatory phenotype markers. Furthermore, all three BCKAs can fuel TCA cycle metabolite pools. KIC and KMV can also increase immunosuppressive metabolites in macrophages. Proteomics and network analysis identified several canonical functions significantly altered by KMV treatment of macrophages, including TNFα-NFκB pathway, FcγR-mediated phagocytosis, apoptosis, and redox regulation. We also provide evidence that BCKAs depend on MCT1. These findings highlight the importance of cancer-derived BCKAs for regulating macrophage polarization and metabolism. Studies focusing on the actual net degree of tumor promotion by BCKA-exposed macrophages and its reliance on observed metabolic changes are warranted, most preferably in an appropriate *in vivo* setting.

## Materials and methods

### Animals

6−8-week-old wild-type C57BL/6JRI mice were purchased from Janvier (France). The animals were kept in a pathogen-free environment. Every procedure was carried out under sterile conditions and according to the regulations of the Ethics Committee for the Care and Use of Laboratory Animals at the Medical University of Vienna.

### Cell culture

All cells were cultured at 37°C in a humidified (5% CO2) atmosphere. All cancer cell lines were obtained by ATCC and routinely cultured in Dulbecco′s Modified Eagle′s Medium (DMEM) high glucose containing 10% heat-inactivated FBS, 2 mM L-glutamine, 100 U/mL penicillin and 100 µg/mL streptomycin (DMEM complete medium). All the cultured cells were tested negative for mycoplasma contamination regularly.

Bone marrow derived macrophages (BMDM) were obtained as previously described (48). Briefly, femurs and tibiae from wild-type C57BL/6JRI mice (Janvier) aged 6 to 8 weeks were flushed and bone marrow (BM) cells collected by centrifugation at 400*g* for 5 min at 4 °C. BM cells were resuspended in BMDM differentiation medium and cultured in non-tissue culture treated petri dishes for 6-7 days. BMDM differentiation medium consists of DMEM high glucose, 10% heat-inactivated FBS, 2 mM L-glutamine, 100 U/mL penicillin and 100 µg/mL streptomycin supplemented with 20% L929-conditioned supernatant. The medium was replaced every 3 days. On day 6 or 7, differentiated BMDM (96% of the cells were positive for F4/80 and CD11b) were washed, harvested, and seeded in the DMEM complete medium for different experiments. iBMDM was kindly provided by Laszlo Nagy (Debrecen University, Hungary) and cultured in iBMDM medium consisting of DMEM high glucose, 10% heat-inactivated FBS, 10% L929-conditioned supernatant, 2 mM L-glutamine, 100 U/mL penicillin and 100 µg/mL streptomycin.

### CM preparation

5 × 10^6^ cancer cells or BMDM or iBMDM were seeded in 20 mL DMEM complete medium, and conditioned medium (CM) was collected after 24 h and centrifuged at 410*g*. for 4 min. The supernatant was passed through a 0.22-µm filter to eliminate debris before use.

### Proliferation assay

Cancer cells were seeded in 24-well plates (AE17 2 × 10^4^ cells per well, A549 4 × 10^4^ cells per well), BMDM or iBMDM in 12-well plates (4 × 10^4^ cells per well) in DMEM complete media and allowed to grow overnight. Macrophages were then washed with PBS and received fresh medium supplemented with or without indicated concentrations of BCKAs. To maintain constant nutrient levels and remove waste liberated from dead cells, the medium was replaced every 24 h. Live cell numbers were quantitated with the Vi-CELL XR cell counter (BECKMAN). KIV (Cat: HY-W006057) and KMV (Cat: HY-113063) were purchased from MedChemExpress. KIC (Cat: 68255) was purchased from Sigma-Aldrich. All BCKAs are made up in DMEM. The experiment was performed in 3 independent biological replicates.

### Metabolomic analysis

Metabolites in the conditioned media (CM) or iBMDM were extracted and analyzed according to previous established method with modifications (48). In brief, iBMDM were seeded at 0.85 × 10^6^ cells per well of a 6-well plate and allowed to attach for overnight. Cells were washed with PBS and received fresh medium supplemented with 200 μM KIV, 200 μM KIC and 200 μM KMV, respectively. After 2 h and 24 h incubation, cells were washed in cooled 0.9% NaCl and extracted in 1 mL/well of 80% methanol with 0.3 nM Pentaerythritol and 2.5 nM Phenyl β-D-glucopyranoside as internal extraction standards. Extraction samples were incubated for 15 min at 4°C, then centrifuged for 10 min at 21,000*g*. The supernatant was transferred to a fresh polypropylene tube and dried in a SpeedVac (Labogene). The cell pellet was lysed by RIPA and used to measure protein levels for normalization purposes. 15 μL of methoxyamine hydrochloride solution (40 mg dissolved in 1 ml pyridine) was added to the dried fraction which was then incubated for 90 min at 30°C. Next, 60 μL of (N-Methyl-N-trimethylsilyltrifluoracetamid) MSTFA was added and incubated for 30 min at 37°C. Reaction mixtures were centrifuged for 10 min and 4°C at 21,000*g* and the supernatants were transferred to glass vials with micro-inserts. Measurement of metabolites was performed using gas chromatography-mass spectrometry (GC-MS) standard protocols (49). Deconvolution of the total ion chromatogram, peak alignment and integration was performed using the software MS-DIAL v4.7 (50).

To measure the KIC, KIV, KMV, leucine, isoleucine, and valine contents on CM samples, three aliquots (100 μL) were run for each CM. Briefly, 400 μL prechilled methanol containing internal standards was added to 100 μL media samples and kept for 1h at 4°C. The samples were then centrifuged for 10 min and 4°C at 21,000*g*, and the supernatants were transferred to fresh tubes and dried in a SpeedVac. 20 μL methoxyamine hydrochloride solution (40 mg/1 mL pyridine) and 80 μL MSTFA were used for metabolite derivatization. Measurement and data process was performed as described above. Different concentrations of standard compounds were extracted and measured under the same conditions to calculate the standard curves for absolute quantification.

The analysis of cellular acetyl-CoA and succinyl-CoA was performed using microflow liquid chromatography in combination with an Orbitrap Elite mass spectrometer (LC-MS/MS, Thermo Fisher Scientific) system according to Neubauer et al. (51) with some modifications. Briefly, iBMDM was seeded at 0.8 × 10^6^ cells per well of a 6-well plate and allowed to attach for overnight. Cells were washed with PBS and received fresh medium supplemented with 200 μM KIV, 200 μM KIC and 200 μM KMV, respectively. After 24 h incubation, cells were washed in cooled 0.9% NaCl and quenched in 80% methanol (-20°C). The quenched cells were scraped and incubated for 30 min at 4°C, then centrifuged for 10 min at 21,000*g*. The supernatant was transferred to a fresh polypropylene tube and dried in a SpeedVac. MS buffer (20 mM NH_4_OAc in mqH_2_O and 2% methanol, pH 6.7) was added to the dried fraction and CoA standards which were then centrifuged for 10 min at 21,000*g* and the supernatant was transferred to LC-MS vials. 5 μL of the sample was injected into a Accucore™ Vanquish C-18+ UHPLC column (100 × 2.1 mm; 1.5 µm particle size), equipped with an Accucore™ Defender guards pk4 guard column (150 - C18 10 × 2.1 mm, 2.5 µm particle size (Thermo Fisher Scientific). The mobile phase system consisted of a mixture of solvent A (20 mM NH_4_OAc in mqH_2_O, pH 6.7) and solvent B (LC-MS grade methanol). A gradient elution method was used for the analysis, 0–1 min 5% B, 1–30 min linear gradient to 85% B, 30-30.1 min 0% B, and 30.1–40 min 0% B. The flow was kept constant at 0.25 mL/min and the column was kept at 30°C throughout the analysis. MS-analysis was performed in positive ion mode with the following parameters: Resolution, 120,000; spray voltage, 3.8 kV; capillary temperature, 350°C; sheath gas, 5; auxiliary gas, 0. The mass scanning range of the MS1 fullscan was set at 350–1200 m/z. The collision energy for collision-induced dissociation (CID) was set at 35 eV. Xcalibur (Thermo Fisher Scientific) and MS-DIAL were used to analyze the data.

### Sample preparation for proteomics analysis

Proteomic analyses were performed according to established protocols with modifications (48). Briefly, iBMDM was seeded in DMEM complete medium and allowed to attach for overnight. Cells were stimulated with AE17 CM, 200 μM KIV, 200 μM KIC and 200 μM KMV, respectively. After 24h incubation, cells were washed and harvested in lysis buffer (6M guanidinium chloride (GdmCl), 100 mM Tris pH 8.5, 10 mM tris-(2-carboxyethyl)-phosphin-hydrochloride (TCEP), 40 mM 2-chloroacetamide(CAA)). Lysates were heated for 5 min at 95°C and sonicated with a tip–probe sonicator at 4°C (3 × 30 s of 1 s on and 1 s off at 80% output power). The protein concentration was determined by a BCA assay and adjusted to a concentration of 0.6 µg/µL. 60 µg of protein solution was diluted with 15% aqueous acetonitrile (ACN), and digested with 200:1 (protein:enzyme) LysC at 37°C for 1 h. Then, 10% aqueous ACN in 25 mM Tris (pH 8.5) was added to obtain a final concentration of 0.5 M GdmCl and a final volume of 1000 µL. Samples were incubated with trypsin 50:1 (protein:enzyme) overnight at 37°C. To stop the digestion process, digested peptides were acidified to a final concentration of 1% Trifluoroacetic acid (TFA). The peptides were desalted with MonoSpin C18 columns according to the manufacturer’s instruction. The peptides were eluted with 2 × 60 µL ACN and concentrated in a SpeedVac for 1 h at 45°C. Finally, they were reconstituted in MS loading buffer (2% ACN, 0.1% formic acid (FA)) for LC-MS/MS analysis. The experiment was performed in 3-4 biological replicates.

### LC-MS-based proteomics

Shotgun proteomics was performed according to established protocols (48). LC-MS/MS runs were performed on the UltiMate 300 system (Thermo Scientific) coupled to a Q-Exactive Plus mass spectrometer (Thermo Scientific) The peptides were separated by reversed-phase chromatography using a binary buffer system consisting of 0.1% FA (buffer A) and 90% ACN with 0.1% FA (buffer B). 2 μg of peptides were loaded on a 50 cm column with a 75 μM inner diameter and 2 μM C18 particles (EASY-spray PepMap, Thermo Fisher) and separated by a 170 min gradient (4-35% buffer B over 110min, 35-90% buffer B over 1 min) at a flowrate of 300 nL/min. MS data were acquired using a data-dependent top-20 method with a maximum injection time of 50 ms, a scan range of 300–1650 m/z, and an AGC target of 3e6. The resolutions of the MS and MS/MS spectra were 70,000 and 17,500, respectively. The AGC for MS/MS acquisition was set to 5e4. The max IT and dynamic exclusion were set to 100 ms and 20s, respectively.

Raw mass spectrometry data were processed with MaxQuant version v2.0.3.1 using the default setting if not stated otherwise (52, 53). Oxidized methionine (M) and acetylation (protein N-term) were selected as variable modifications, and carbamidomethyl (C) as fixed modifications. Three missed cleavages for protein analysis were allowed. Label-free quantitation (LFQ) and “Match between runs” were enabled. Searches were performed against the mouse UniProt FASTA database (March 2021) containing 22,001 sequences. Bioinformatics analysis was performed using Perseus v1.6.6.0 (54) and COVAIN toolbox (55). The proteinGroups output table was used for all proteomic analyses. Reverse proteins, proteins that were only identified by site, and potential contaminants were filtered out. The protein groups were filtered to have at least 70% valid values, reaching a list of 3512 protein groups (**Table S1**), which were further used for all downstream analyses. Dataset integration was based on gene name. Missing values were imputed using a minimal value model within the COVAIN toolbox. Significantly up-or-downregulated proteins (DEPs) between the three BCKAs groups and the control group were determined by ANOVA (FDR < 0.05) and used for all downstream analyses.

### Enrichment analysis

Pathway analysis of metabolomics data was performed using the online tool MetaboAnalyst v5.0 (56). Enrichment analyses of proteomics data were performed using the Cytoscape v3.8.2 (57) module ClueGO v2.5.8 (58). Enrichments were performed using the GO, KEGG, REACTOME and Wiki Pathway databases. Only pathways with *p* < 0.05 and at least a 4 gene overlap were considered for grouping (kappa score 0.4). A protein network was generated with DEPs using STRING v11.5 (59) and selected KEGG pathways (FDR < 0.05) were highlighted.

### Cytokine production measurement

Differentiated BMDMs were seeded at a density of 0.9 × 10^6^ cells per well in 6-well plates in 2 mL of DMEM complete medium overnight before the media was either replaced with AE147 CM or was supplemented with 200 μM KIV, 200 μM KMV, 200 μM KIV or BCKA pool. After 24 h, cell-free supernatants were collected. The levels of IL-6 (Sigma, RAB0308-1KT), TNF-α (Sigma, RAB0477-1KT), TGF-β (Invitrogen, BMS608-4) and VEGF (Sigma, RAB0509-1KT) in the supernatants were measured by ELISA kits according to the manufacturer’s instructions. The experiment was performed in 3 biological replicates.

### Immunoblotting

BMDM or iBMDM were lysed on ice in self-made RIPA lysis buffer supplemented with a proteinase inhibitor cocktail (Sigma; 4693116001). After 10 min incubation on ice the lysates were subjected to sonication with a tip–probe sonicator at 4°C (3-5 s of 1 s on and 1 s off at 80% output power) to effectively shear DNA and reduce viscosity. After centrifugation, the pellet was discarded and the protein concentration in the supernatant was measured by a BCA assay. Equal amounts of denatured lysate were separated by 10% SDS-PAGE and transferred to PVDF membranes. Membranes were blocked in 5% low-fat milk for 1 h at room temperature (RT) and incubated with primary antibodies at 4°C overnight. Membranes were then incubated with HRP-conjugated secondary antibodies for 1 h at RT. The signal was detected using the ECL system (Amersham Biosciences, Cytiva) according to the manufacturer’s instructions (iBright FL1500 Imaging System, Thermo Fisher). The primary antibodies used were against ARG1 (Santa Cruz; sc-47715) and α-tubulin (Proteintech; HRP-66031). Appropriate secondary antibodies were from Proteintech. The experiment was performed in 3 biological replicates.

### RNA isolation and gene expression analysis

BMDM or iBMDM were washed twice and suspended directly in TRI reagent (Thermo Scientific; 15596026). Total RNA was isolated according to the manufacturer’s instructions. Quality and quantity were measured on a Nanodrop (Thermo Scientific). cDNA was synthesized from 1 μg of RNA using the GoScriptTM Reverse Transcription kit (Promega). mRNA levels were determined using the Luna Universal qPCR Master Mix (New England biolab) on a Bio-Rad CFX Connect. The quantification of the results was performed by the comparative Ct (2–ΔΔCt) method. The Ct value for each sample was normalized to the value for the *Rps9* gene. The following primer pairs were used:*Arg1*, ACATTGGCTTGCGAGACGTA, ATCGGCCTTTTCTTCCTTCCC; *Mrc1*, CTCTGTTCAGCTATTGGACGC, CGGAATTTCTGGGATTCAGCTTC; *Rps9*, GCAAGATGAAGCTGGATTAC, GGGATGTTCACCACCTG; *Nos2*, CAGAGGACCCAGAGACAAGC, TGCTGAAACATTTCCTGTGC; *Vegf*, GGCCTCCGAAACCATGAACT, CTGGGACCACTTGGCATGG; *Mgl1*, TGCAACAGCTGAGGAAGGACTTGA, AACCAATAGCAGCTGCCTTCATGC; *Mgl2*, GCATGAAGGCAGCTGCTATTGGTT, TAGGCCCATCCAGCTAAGCACATT; *Il6*, ACAAAGCCAGAGTCCTTCAGAGAG, TTGGATGGTCTTGGTCCTTAGCCA; *Il1β*, TGGCAACTGTTCCTG, GGAAGCAGCCCTTCATCTTT; *Slc16a1*, GGGCTAAAGCCACAGTCCAT, TCTGCTAAGTGCCACACAGG. The experiment was performed in 4 independent biological replicates.

### Quantification and statistical analysis

Statistical significance was calculated between two groups by student’s unpaired t-test. One-way ANOVA with Tukey’s HSD post-test was used to calculate statistical significance between multiple groups. Analyses were performed using Microsoft Excel or GraphPad Prism v9. Error bars represent SEM and *p* < 0.05 was considered statistically significant (∗*p* < 0.05, ∗∗*p* < 0.01, ∗∗∗*p* < 0.001).

## Data availability statement

The mass spectrometry-based proteomics data have been deposited at ProteomeXchange Consortium (http://www.proteomexchange.org/) with the accession number PXD032100. Metabolomics data have been deposited to the EMBL-EBI MetaboLights database (https://www.ebi.ac.uk/metabolights) with the identifier MTBLS4497.

## Ethics statement

The animal study was reviewed and approved by Ethics Committee for the Care and Use of Laboratory Animals at the Medical University of Vienna.

## Author contributions

ZC and WW conceived and designed the study; ZC and WL performed the experiments; MB and SB helped with instruments and techniques; EH provided necessary tools, supervised, and supported experiments; ZC, WL, and WW wrote the manuscript. All the authors read, revised, and agreed on the final version of the manuscript.

## Funding

ZC and WL were supported by Ph.D. scholarships provided by China Scholarship Council (CSC) (Grant numbers: 201806500012 and 201908320480). Part of this work was supported by the Austrian Science Fund (FWF; grant number P32600 to EH).

## Acknowledgments

We thank Petra Heffeter (Medical University of Vienna, Austria) for providing the C57BL/6JRI mice. We thank Leila Afjehi, Palak Chaturvedi and Florian Schindler (University of Vienna, Austria) for mass spectrometry assistance. We thank Shomaila Mehmood (Anhui University, China) for helpful discussions.

## Conflict of interest

The authors declare that the research was conducted in the absence of any commercial or financial relationships that could be construed as a potential conflict of interest.

## Publisher’s note

All claims expressed in this article are solely those of the authors and do not necessarily represent those of their affiliated organizations, or those of the publisher, the editors and the reviewers. Any product that may be evaluated in this article, or claim that may be made by its manufacturer, is not guaranteed or endorsed by the publisher.

## Supplementary material

Supplementary Figure 1 BCKAs in cancer cell CM induce murine iBMDM polarization. **(A)**, Schematic showing how branched-chain amino acids (BCAAs) (leucine, isoleucine, and valine) are broken and oxidation. BCAA transaminases 1 and 2 (BCAT1/2) transfer nitrogen to a-ketoglutarate (a-KG) to produce glutamine and the specifed BCKAs, which then metabolized by branched-chain a-keto acid dehydrogenase (BCKDH) complex to produce a branched-chain acyl-CoA (R-CoA) that can be further metabolized to the TCA cycle intermediates acetyl-CoA or succinyl-CoA. **(B)**, Relative proliferation rate of iBMDM grown in DMEM complete medium or indicated cancer cell CM. **(C)**, Relative proliferation rate of BMDM grown in DMEM complete media or equivalent media containing 200 μM KIV or 200 μM KIC or 200 μM KMV (n = 3). **(D)**, mRNA expression of *Arg1*, *Mrc1*, *Mgl1* and *Mgl2* in iBMDM was measured by qPCR after incubation with AE17 CM for 24 hours. **(E)**, ARG1 protein level was measured by western blot after incubation with AE17 CM for 24 hours. **(F)**, mRNA expression of *Arg1*, *Vegf*, *Il6*, *Nos2* and *Slc16a1* was measured in iBMDM after stimulation with DMEM (Control), 200 μM KIV, 200 μM KIC and 200 μM KMV, respectively for 24 hours. **(G)**, mRNA expression of *Arg1* and *Vegf* in iBMDM was measured after pretreatment with the MCT1 inhibitor 250 nM AZD3965 for 1 hour, and stimulation with 200 μM KIV, 200 μM KIC and 200 μM KMV, respectively for 24 hours. Data show the mean ± SEM of n = 3 biological experiments. ∗*p* < 0.05, ∗∗*p* < 0.01, ∗∗∗*p* < 0.001 (unpaired two-tailed t test).

Supplementary Figure 2 Protein signatures of responses to BCKAs stimulation, related to **(A)**, Principal Component Analysis (PCA) of proteins shown in **(B)**, Selected functional categories (GO, KEGG, REACTOME and Wiki Pathway databases) of BCKAs-stimulated iBMDM versus unstimulated iBMDM. BCKAs upregulated proteins are enriched cAMP signaling pathway, sphingolipid signaling pathway, autophagy, VEGF signaling, purine nucleotide biosynthesis process (left). BCKAs downregulated proteins are enriched in RNA splicing, redox regulation, apoptosis, NF-κB pathway (right). PCA plot was applied *via* toolbox COVAIN (55).

Supplementary Figure 3 BCKAs stimulation reprogrammed macrophage metabolism. **(A)**, Glutamine and glutamic acid levels after individual BCKAs treatment were shown. **(B),** Pathway analysis of 17 metabolites (FDR *p* < 0.05, one-way ANOVA) between control and three BCKA groups at 24 h. First 3 critical pathways are highlighted.
