# Supplementary figures and images for: Branched-chain ketoacids derived from cancer cells modulate macrophage polarization and metabolic reprogramming
Source: Front Immunol. 2022 Oct 13;13:966158. doi: 10.3389/fimmu.2022.966158 (PMC9606345; doi:10.3389/fimmu.2022.966158)

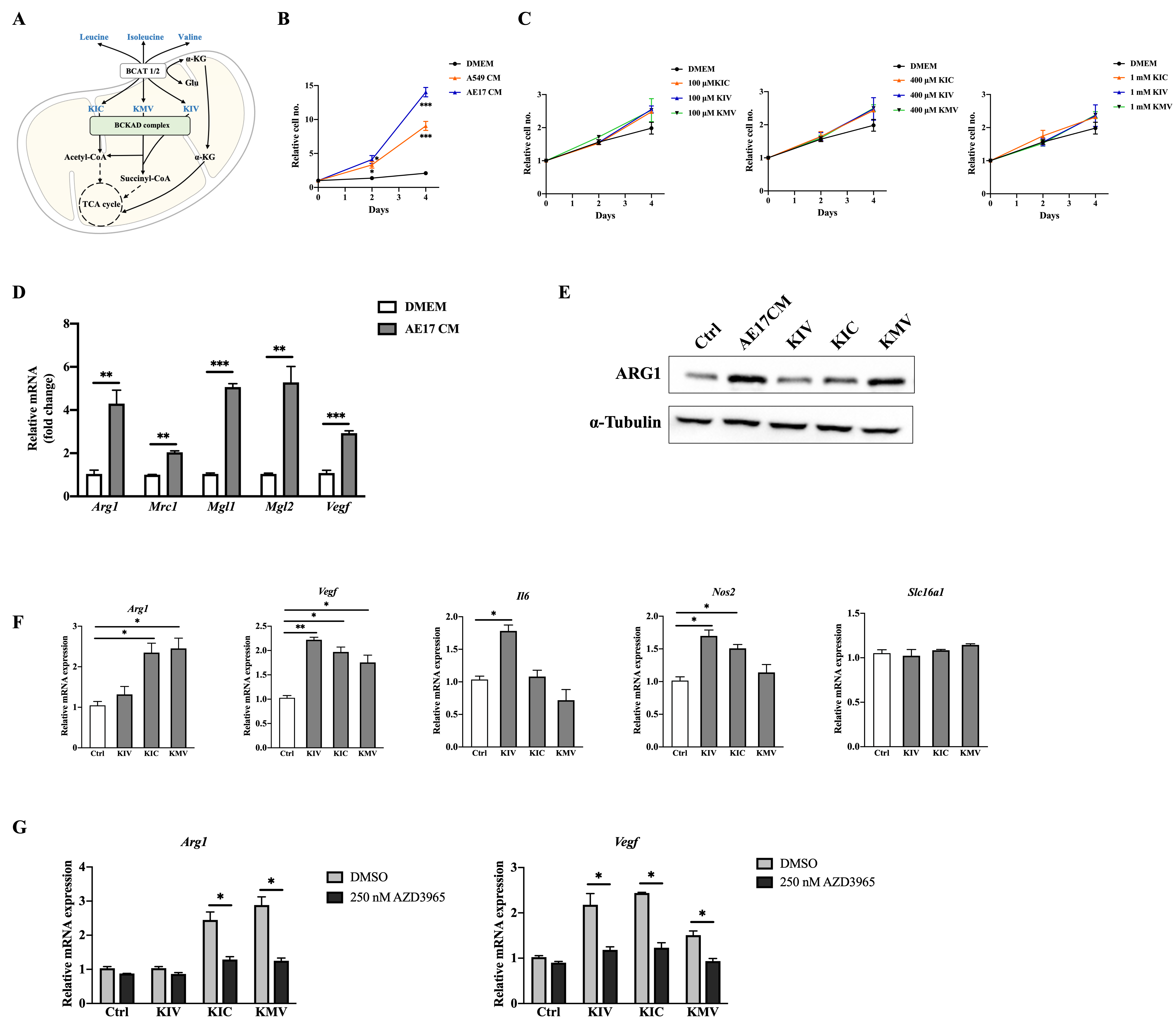

Supplement: Supplementary Figure 1 — BCKAs in cancer cell CM induce murine iBMDM polarization. (A), Schematic showing how branched-chain amino acids (BCAAs) (leucine, isoleucine, and valine) are broken and oxidation. BCAA transaminases 1 and 2 (BCAT1/2) transfer nitrogen to a-ketoglutarate (a-KG) to produce glutamine and the specifed BCKAs, which then metabolized by branched-chain a-keto acid dehydrogenase (BCKDH) complex to produce a branched-chain acyl-CoA (R-CoA) that can be further metabolized to the TCA cycle intermediates acetyl-CoA or succinyl-CoA. (B), Relative proliferation rate of iBMDM grown in DMEM complete medium or indicated cancer cell CM. (C), Relative proliferation rate of BMDM grown in DMEM complete media or equivalent media containing 200 μM KIV or 200 μM KIC or 200 μM KMV (n = 3). (D), mRNA expression of Arg1, Mrc1, Mgl1 and Mgl2 in iBMDM was measured by qPCR after incubation with AE17 CM for 24 hours. (E), ARG1 protein level was measured by western blot after incubation with AE17 CM for 24 hours. (F), mRNA expression of Arg1, Vegf, Il6, Nos2 and Slc16a1 was measured in iBMDM after stimulation with DMEM (Control), 200 μM KIV, 200 μM KIC and 200 μM KMV, respectively for 24 hours. (G), mRNA expression of Arg1 and Vegf in iBMDM was measured after pretreatment with the MCT1 inhibitor 250 nM AZD3965 for 1 hour, and stimulation with 200 μM KIV, 200 μM KIC and 200 μM KMV, respectively for 24 hours. Data show the mean ± SEM of n = 3 biological experiments. ∗p < 0.05, ∗∗p < 0.01, ∗∗∗p < 0.001 (unpaired two-tailed t test). [file Image_1.tiff]

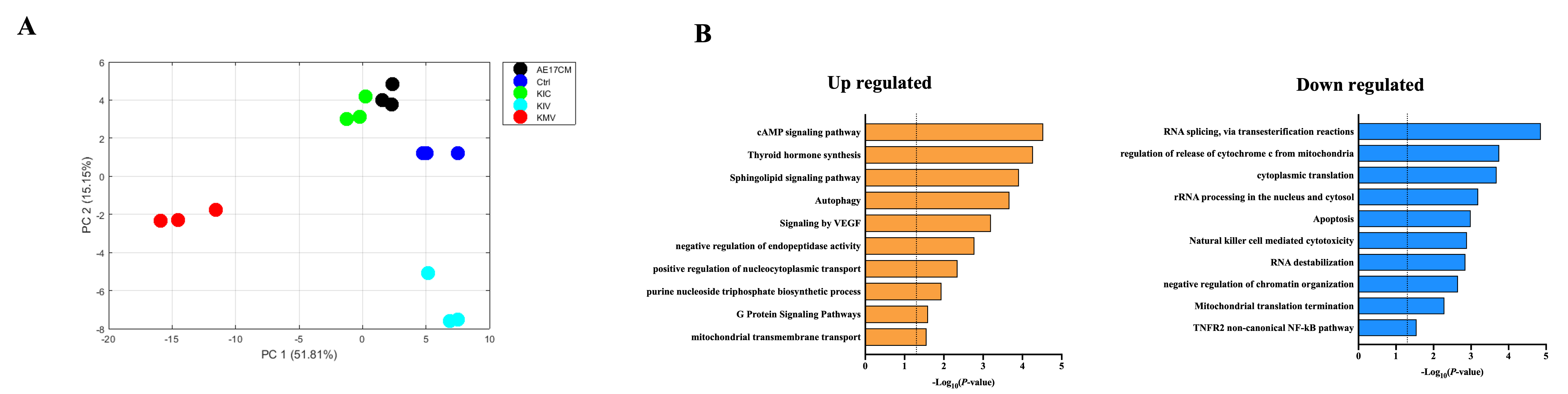

Supplement: Supplementary Figure 2 — Protein signatures of responses to BCKAs stimulation, related to (A), Principal Component Analysis (PCA) of proteins shown in (B), Selected functional categories (GO, KEGG, REACTOME and Wiki Pathway databases) of BCKAs-stimulated iBMDM versus unstimulated iBMDM. BCKAs upregulated proteins are enriched cAMP signaling pathway, sphingolipid signaling pathway, autophagy, VEGF signaling, purine nucleotide biosynthesis process (left). BCKAs downregulated proteins are enriched in RNA splicing, redox regulation, apoptosis, NF-κB pathway (right). PCA plot was applied via toolbox COVAIN (55). [file Image_2.tiff]

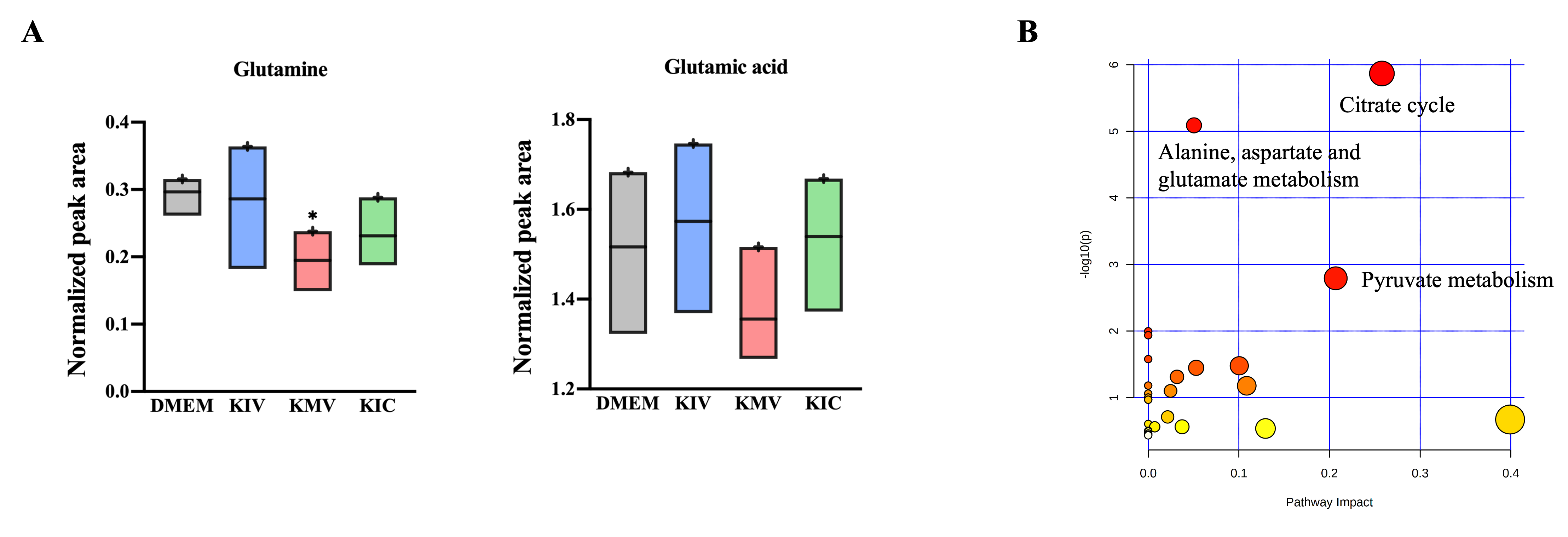

Supplement: Supplementary Figure 3 — BCKAs stimulation reprogrammed macrophage metabolism. (A), Glutamine and glutamic acid levels after individual BCKAs treatment were shown. (B), Pathway analysis of 17 metabolites (FDR p < 0.05, one-way ANOVA) between control and three BCKA groups at 24 h. First 3 critical pathways are highlighted. [file Image_3.tiff]
